# Velpeau's Anatomy of Regions

**Published:** 1838-07

**Authors:** 


Art. II.—Velpeau's *Anatomy of Regions. Translated from the French.* By Henry Hancock, Lecturer on Practical and Surgical Anatomy at the Westminster Hospital School of Medicine, &c.—*London*, 1838. 8vo. pp. 565.

The object of the translator in adapting the original treatise to the English reader has been "to furnish the student with a complete work on the anatomy of regions, restrained within such limits as would make it available to all members of the profession." For this purpose, he has omitted the portion of the original which treats of general anatomy, and also the plates. The merits of M. Velpeau's work are too well known to require eulogy from us; but we may be permitted to doubt whether, by translating it in full, Mr. Hancock has presented to the English student exactly the book which he requires. There is in this, as in many of the French anatomical works, an unnecessary minuteness of detail, which is, in a practical view, not only superfluous but absolutely injurious. It is not to be expected that any one should retain in his memory all the multitude of insulated facts enumerated in the work before us; and we cannot help thinking that, in attempting to learn too much, the student is liable to have his mind distracted from topics of more real importance. No surgeon can be too well acquainted with the detailed anatomy of those regions which contain parts whose distribution and arrangement may affect his treatment of injury or disease; but, to map out the whole body by lines drawn from its external prominences, and to investigate the collocation of every organ and every tissue contained in each division, whether practically important or not, seems to us a labour which should not be imposed on any but those who are to make anatomy the study of their lives.

We cannot give Mr. Hancock by any means unqualified praise for the execution of the translation. Plain statements of facts cannot be too simple and clear; and we regret to say that, whether from following his original too closely, or from his own want of practice as a writer, Mr. H. not only makes occasional use of idiomatic phrases foreign to our language, but even falls into errors of construction and style. Thus, we find in the first page the following sentence: "increasing in thickness, as *it* [the skin] proceeds, posteriorly, from the ribs; *it* thus explains why furuncula and anthrax are so frequent on the back." And, in the next page, we have a short paragraph of five lines and a half containing seventeen commas, unrelieved by any other stop. On the whole, however, the book is a valuable one, and will be useful to many who are not masters of the original.

A concise description of the anatomy of the regions concerned in surgical operations or peculiar local diseases, illustrated by good but cheap delineations, and leaving out of view all but directly practical objects, seems to us still a desideratum in our medical literature.

